# Risk of Exposure to *Coccidioides* spp. in the Temblor Special Recreation Management Area (SRMA), Kern County, CA

**DOI:** 10.3390/microorganisms11020518

**Published:** 2023-02-17

**Authors:** Antje Lauer, Jocelyne Jassiri Lopez, Michael Chabolla, Carl Kloock

**Affiliations:** Department of Biology, California State University Bakersfield, 9001 Stockdale Highway, Bakersfield, CA 93311, USA

**Keywords:** arthroconidia, BLM, best management practices, *Coccidioides*, diagnostic PCR, dust emissions, Valley fever, Temblor Mountains, off-road vehicles, pathogen exposure risk, recreation soil analyses

## Abstract

The Temblor Mountain Special Recreation Area (SRMA) on the western flank of the San Joaquin Valley, CA, is located in the endemic area of *Coccidioides,* a fungal pathogen responsible for the increasing incidence of coccidioidomycosis (Valley fever). Recreationists in the SRMA, such as off-highway vehicle (OHV) drivers and mountain bikers who disturb the soils, are at risk of being exposed to airborne arthroconidia (asexual spores) of the pathogen. To reduce the risk of pathogen exposure for visitors, the Bureau of Land Management (BLM) plans to limit recreational activities to areas with a reduced pathogen presence. They envision an official OHV park in the future, by also restricting access to areas with ongoing restoration efforts and by limiting soil erosion in sensitive areas. To investigate which soils in the Temblor SRMA are most likely to support the growth of *Coccidioides* spp., soil samples were collected over a 3-year period from dominant soil types in a northern and a southern sampling area and analyzed for the pathogen using a culture-independent PCR-based method. In addition, soil pH and electrical conductivity were determined. The results of this study revealed slight genetic variance in the *Coccidioides* sequences obtained from the soils of the Temblor SRMA. An analysis of variance (ANOVA) could not confirm differences in soil pH and electrical conductivity (EC) between the different soil types investigated and between sites where the pathogen was detected compared to sites where it could not be found. However, the year of sampling appeared to have an influence on observed soil pH and EC, and the presence of the pathogen. Of all dominant soil types investigated, those belonging to the Littlesignal–Cochora association were the least likely to contain the pathogen, whereas soils of the Beam–Panoza–Hillbrick association appeared more supportive. In addition to pointing out OHV areas with lower pathogen exposure risk in the Temblor SRMA, recommendations were made to educate visitors and BLM workers about the risk of contracting Valley fever.

## 1. Introduction

The Temblor Special Recreational Management Area (SRMA), newly designated in December 2014, and managed by the Bureau of Land Management (BLM), comprises 25,688 acres located between the San Joaquin Valley and the Carrizo plains in western Kern County and eastern San Luis Obispo County in California. The soils are generally shallow, well-drained sandy loams, which formed from weathered sandstone or diatomaceous shale (Taxonomic class: Loamy-skeletal, mixed, superactive, thermic Lithic Haploxerolls) [1]. The mountain range is used for livestock grazing, watershed, and wildlife habitats. Furthermore, the hilly and rocky terrain is frequented year-round by off-highway vehicle (OHV) riders, recreational hunters, shooters, and hikers. However, even though there is limited legal access to the SRMA, many visitors trespass into the area, ignoring signs indicating where restoration efforts for native plants are in progress and where field workers have fenced off erosion-damaged hillsides.

In addition, many visitors do not know about the risk of contracting Valley fever. Arthroconidia (asexually produced spores) of the pathogen *Coccidioides* spp. in fugitive dust can emerge from OHV activities, especially during the dry season. Valley fever is caused by the soil-borne dimorphic fungi *C. immitis* which is endemic to California, and by *C. posadasii* which is established in arid and semi-arid regions of southwestern U.S., Mexico, and in some areas of South America [2,3]. Although still considered an orphan disease, the reported incidence of Valley fever is on the rise and has been documented by epidemiological studies and supported by data available from the California Department of Public Health online data portal [4,5]. Symptoms of the disease often mimic other infectious and non-infectious diseases (Valley fever is sometimes called desert rheumatism) which can lead to misdiagnosis and delayed treatment. The misdiagnosis of Valley fever can result in the dissemination of the disease, which often requires life-long treatment and can even be lethal for some patients [6], resulting in extensive healthcare and economic burdens [7,8,9,10].

Although the Temblor Mountains are in the endemic area of *C. immitis*, the dominant pathogen in California, previous work focusing on detecting *Coccidioides* in the soils of the SRMA has not occurred. Measurements of soil pH and electrical conductivity were part of the soil analysis of all sampling sites, as well as a documentation of the dominant plants and evidence of rodent activity. Previous work on the detection of *Coccidioides* in the soils of Kern County found several soil parameters to be indicative of a suitable habitat for the pathogen, such as an elevated pH, increased electrical conductivity, and a clay content of about 20–30% [11,12]. The presence of rodents is thought to be supportive for keratinophiles, such as *Coccidioides* and other members of the fungal order Onygenales. The keratin in feces from skin and fur might attract and facilitate the growth of the pathogen [13,14].

The major aim of this study was to reduce health hazards to all visitors in the Temblor SRMA. The BLM Bakersfield Field Office is considering proposing an official OHV park in an area where the risk of pathogen exposure is low, guiding visitors away from areas with high exposure risk and reducing access to areas where restoration efforts are ongoing. To achieve this task, a rigorous soil sampling plan was designed to identify specific soil types in the SRMA as potential sources of *Coccidioides* arthroconidia that visitors could be exposed to when soil is disturbed. Soil samples from dominant soil types in the Temblor SRMA that differ in terms of soil parent material, and chemical and physical parameters, were collected and analyzed for the presence of *Coccidioides* over several years using a culture-independent nested Polymerase Chain Reaction (PCR)-based approach including a diagnostic PCR step that is specific to the genus *Coccidioides*. Based on the results of this study, recommendations were communicated to the BLM Bakersfield Field Office, indicating areas with low and high *Coccidioides* exposure risks.

## 2. Material and Methods

### 2.1. Sampling Plan

A soil sampling plan was developed based on recommendations described by Wollum (1994) [15]. The United States Department of Agriculture (USDA) websoilsurvey (WSS) tool was used to identify the dominant soil types present in the Temblor SRMA. Based on this assessment, two representative areas, one in the southern SRMA, entering at Hill Road, Chevron Entrance 15B/C, and one in the northern SRMA, entering at Molca Street, Chevron Entrance 40, were chosen to collect unique soil samples from all dominant soil types. Both areas are frequented by off-road vehicles during all seasons, the southern area more frequently than the northern area. Not all sites were accessible because of the poor road conditions damaged by weather events during the wet season. Soil sampling was performed by following accessible trails in the area, and randomly collecting samples along the trail. A characterization of soil microbiological or biochemical properties is challenging and can at best represent a snapshot of a current environmental situation, which can change seasonally and with soil depth. However, for practical reasons, the sampling size was limited and not all microhabitats in an area were investigated.

### 2.2. Sampling and Sample Storage

Soil samples were collected in November 2018, June 2019, and March 2021, following the same sampling trails whenever accessible. Sampling sites included vegetated and non-vegetated areas and sites that differed with respect to soil disturbance, grazing, and rodent activity. Soil samples were collected aseptically in 50 mL Falcon tubes (5–10 cm (about 4 in) depth, ~40 g). In the northern sampling area, samples were collected along T4300, and T40-4200, and in the southern sampling area soils were collected along T4500, T4200, and T4900. All individual sampling sites were briefly described and photographed. Once in the lab, all samples were frozen at −20 °C until processed.

### 2.3. Safety

All samples were collected safely and comprehensively by following the safety and laboratory protocols developed for this project. We follow the guidelines outlined by the Environmental Protection Agency (EPA) regarding the sampling of soil that potentially includes bacterial pathogens [16].

When sampling soils in *Coccidioides* endemic areas, we avoided fieldwork on predicted windy and dusty days. All practices for Biosafety Level 2 were followed. Researchers wore N95 dust masks in the field when necessary. Soils were handled under a Biosafety Cabinet (LabConco Purifier Class II) and all students involved in this project were instructed to follow the laboratory guidelines used in a standard microbiology lab. Any waste resulting from the DNA extraction procedures was safely stored and later collected by the California State University Bakersfield (CSUB) stockroom technician responsible for waste management. Any soil samples that tested positive for the pathogen were autoclaved for two hours before being properly discarded.

### 2.4. Soil Parameters

The United States Department of Agriculture (USDA) Websoilsurvey (WSS) database was used to assess a selection of soil environmental parameters (depth 0–30 cm), as well as soil types [17]. Individual samples were not bulked prior to measurements.

In addition, soil pH and electrical conductivity (EC, as deciSiemens/cm or mSiemens/cm) were determined in the lab, using a pH meter (Oakton, model 510) and a conductivity/TDS meter (Hach, model 44600), respectively. Soil slurries were mixed with 5 g of soil and 20 mL of distilled water and allowed to stand overnight before measurements were taken.

### 2.5. DNA Extraction and PCR

DNA was extracted from soil samples and biological samples using the DNEasy Powerlyzer DNA extraction kit (Qiagen, Germantown, MD, USA) following the protocol provided by the manufacturer. Cell lysis was enhanced by adding a Proteinase K (100 μg/mL) incubation step at 56 °C for 20 min prior to DNA extraction.

To detect *Coccidioides*, a nested polymerase chain reaction (PCR) method originally published by [18] was used, with modifications to the diagnostic PCR step, where the *Coccidioides* specific primer pair EC3/EC100 was used [19], instead of the primer pair in the original protocol. A nested PCR increases the likelihood of detecting the pathogen in samples where the fungus is not a dominant member of the soil microbial community at the time of sampling. A positive control used for all PCRs was obtained from the non-pathogenic *C. posadasii* Δchs5 strain (NR4548, BEI Resources). The success of DNA extraction and PCR was verified via electrophoresis using 2% agarose gels in 1× Tris-Borate-EDTA (TBE) buffer. Gels were visualized using an SYBR SafeTM DNA stain (Invitrogen, Carlsbad, CA, USA) and documented on a GelDoc System (BioRad, Hercules, CA, USA). All PCRs were performed in duplicate. All PCR products obtained with the diagnostic primer pair were sequenced (Laragen Inc., Culver City, CA, USA) and compared to entries in the GenBank nucleotide database [20].

### 2.6. Statistics

pH and electrical conductivity (EC) data were analyzed separately, but followed the same basic pattern. First, data were aggregated by soil type and year, with any soil types represented by less than 5 samples being eliminated from the data set. Deviations for each were calculated, and the distributions of these deviations were analyzed for normality using distribution plots and quartile–quartile (Q-Q) plots. Non-normally distributed data were successfully normalized by natural log transformation. Next, two-factor ANOVA with year and soil type as factors was conducted to determine if data could be pooled across soils and/or years. This was deemed preferable to simply conducting a three-factor ANOVA with the addition of *Coccidioides* presence/absence as a factor because several site/year combinations yielded ≤1 *Coccidioides*-positive samples, so pooling data where appropriate allows larger sample sizes and a more reliable analysis. For both pH and EC data, this analysis supported pooling across sites but not across years.

Once data were pooled appropriately based on the results of this analysis, one factor ANOVA were conducted with pathogen presence/absence as the independent variable to determine if any consistent differences between soils with *Coccidioides* present or absent in either pH or EC could be detected. All ANOVA were performed using the R Core Team Statistical Software, version 3.1.0 [21].

### 2.7. Evolutionary Relationships of Taxa

In preparation for a phylogenetic comparison of PCR amplicons obtained with the diagnostic primer pair EC3/EC100, a sequence alignment was conducted using MEGA11 [22]. In addition to the sequences obtained in this study, close matches from the GenBank database were added including type strains and a few environmental sequences. The evolutionary history was inferred using the neighbor-joining method with all sequences having the same length. A bootstrap test (1000 replicates) was used and the percentage of replicate trees in which the associated taxa clustered together are shown next to the tree branches. The evolutionary distances were computed using the maximum composite likelihood method and are in the units of the number of base substitutions per site [23,24,25].

## 3. Results

### 3.1. The Temblor SRMA: Soil Types and Landscape

**Figure 1:**
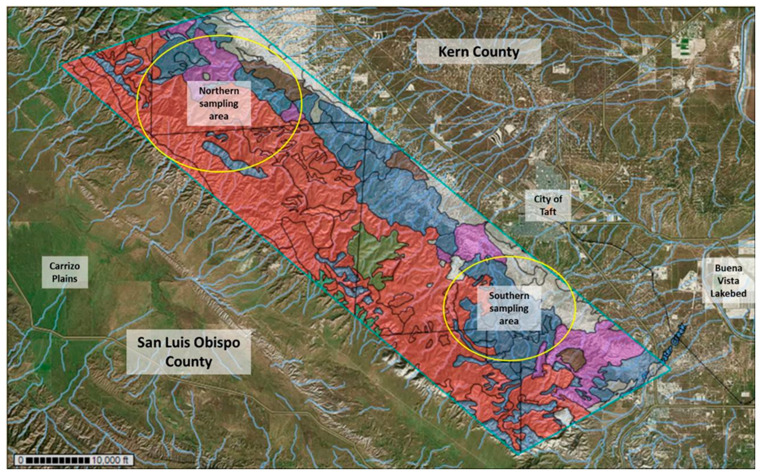
Map of the Temblor SRMA extending over parts of Kern County and San Luis Obispo County, CA, (county border seen as black zigzag line) with indication of the northern and southern sampling areas (yellow circles). Different soil types are indicated in distinct colors based on information from the USDA WSS database (Beam–Panoza–Hillbrick complex (red), Pyxo–Cochora–Badlands association (dark blue), Xeric Torriorthents–Badlands (light blue), Littlesignal–Cochora association (magenta), Reward channery loam (kaki), Elkhills–Welport association (grey), Elkhills–Pyxo association (brown), Padres sandy loam (green). Soils of some less abundant soil types, e.g., soils of the Xerorthents–Badlands association, are not seen in this overview.

The central and western areas of the Temblor SRMA are dominated by soils of the Beam–Panoza–Hillbrick association making it the most dominant soil type in the area. The eastern foothills of the SRMA include large areas of soil of the Pyxo–Cochora–Badlands association, Littlesignal–Cochora association, and Elkhills–Welport association. Other soil types in the area that were less dominant included soils of the Elkhills–Pixo association, Pixo–Kimberlina–Cochora association, Reward–Hillbrick association, as well as Xeric Torriorthents–Badlands, Reward Channery loam, Guijarral gravelly sandy loam, and Padres sandy loam. Based on this information, two large sampling areas were chosen, a southern location and a northern location which included all major soil types. The same dominant soil types were encountered in the northern and southern sampling area; however, soils of the Pyxo–Cochora association were clustered in the center of the southern sampling area, whereas in the northern sampling area, soils of the Beam–Panoza–Hillbrick association prevailed (Figure 1).

Overall, soils of the Beam–Panoza–Hillbrick association appeared to be the most dominant in the Temblor SRMA, expanding over the western and part of the central section of the mountain range, especially in the northern sampling area. In the southern sampling area, soils of the Pyxo–Cochora association that stretch along the eastern lower elevations of the Temblor SRMA are abundant and are also common in the central Temblor Mountain range. Another dominant soil type that relates to soils of the Elkhills–Welport association dominated the eastern foothills of the Temblor SRMA. Samples from several patches of soil associated with the Littlesignal–Cochora soil complex were collected as well and were included in this study. Supplementary Figure S1 gives an impression of the hilly terrain of the southern and northern sampling areas that were accessed.

### 3.2. Sampling

**Figure 2:**
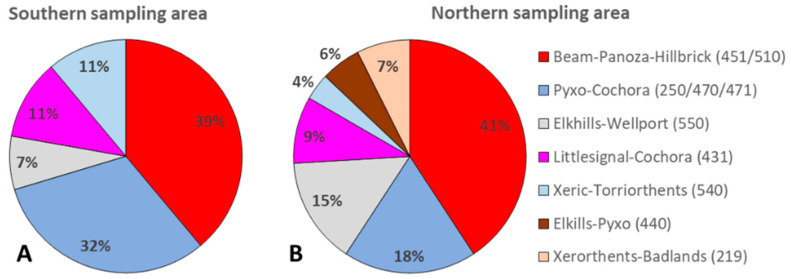
Percentage of soil samples collected from different dominant soil types in the southern (**A**) and northern (**B**) sampling area of the Temblor SRMA between 2018 and 2021 (excluded are soil types that were only sampled occasionally [n = 8]) (overall: n = 106).

Overall, 114 soil samples were collected, with 41 collected in 2018 (southern area n = 23; northern area n = 18), followed by 29 samples in 2019 (southern area n = 10; northern area n = 19), and 44 samples in 2021 (southern area n = 24; northern area n = 20). Soil samples were collected from representative soil types in the area as indicated by the USDA WSS database. Most of the collected soil samples belonged to soils associated with the Beam–Panoza–Hillbrick complex (n = 44 [37.72%]), the most dominant soil type in the target area, and soils of the Pyxo–Cochora association (n = 27 [23.68%]). Additional soil samples belonged to the Elkhills–Wellport association (n = 12 [10.53%]), the Littlesignal– Cochora association (n = 11 [9.65%]), and the Xeric–Torriorthents–Badlands association (n = 8 [7.02%]). Additional soil samples were collected from soils of the Elkhills–Pyxo association (n = 3 [2.63%]) and the Xerorthents–Badlands complex (n = 4 [3.51%]) (both in the northern area only) (Figure 2). Soil samples belonging to some soil types that were less dominant along the trails, e.g., Padres loamy sand, Polonio clay loam, Guijarral gravelly sandy loam, soils of the Pyxo–Kimberlina–Cochora association and Reward–Hellbrick association were analyzed but excluded from graphs and statistical evaluations. In the northern sampling area, soil samples were collected from locations between 1457 and 2720 feet (about 829 m), whereas in the southern sampling area, soil samples were collected at sites between 1483 and 2484 feet (about 757 m).

We attempted to target the same sampling area each year. However, not all areas were accessible each year due to the erosion of some trails. Three trails were followed in the southern sampling area and two in the northern sampling area. In addition to Figure 1, which shows the sampling area that was targeted in all three years, Supplementary Tables S1–S3 combine coordinates, elevation, soil type, and a brief description of all sampling sites.

### 3.3. Environmental Parameters

**Figure 3:**
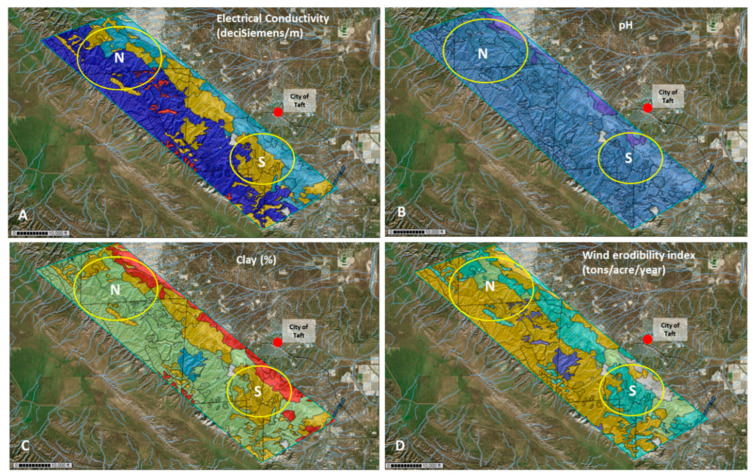
(**A**) Soil electrical conductivity (EC) (deciSiemens/m): 2.6 (dark blue), 2 (light blue), 1.2 (light green), 1 (yellow), 0–0.5 (red). (**B**) Soil pH: 8.6 (dark blue), 7.9–8.2 (blue). (**C**) Percent clay: 21.5 (light blue,) 16.3–18 (light green), 11.3–12 (red), 13.0 (yellow). (**D**) Wind erodibility index (Rating tons per acre per year): 86 (yellow and light green), 56 (turquoise), 38 (dark blue). The southern and northern sampling area are indicated by a yellow circle (S and N). The city of Taft is indicated by a red circle east of the Temblor SRMA.

The USDA WSS database revealed that soil types found in the Temblor SRMA differ in soil physical and chemical parameters, such as parent material, electrical conductivity, pH, and grain size, which is linked to the soil erosion potential. Soil pH in the area of interest was predicted as alkaline for most sites included in this study and ranged between pH 7.9 and 8.2. Electrical conductivity values ranged from low (0–0.5 dS/m) along the eastern foothills to high (up to 2.6 dS/m) in the central and eastern part of the Temblor SRMA. A patch of higher clay content (21.3%) was indicated for a central area of the SRMA, characterized as Padres sandy loam. Other soils investigated in this study contained less clay (<20%), as indicated by the WSS database. The erosion potential of soil types also differed, ranging from 38 tons/acres/year for Padres sandy loam to as high as 86 tons/acres/year for many soils in the central and eastern part of the Temblors, such as the soils of the dominant Beam–Panoza–Hillbrick association. The soil sampling plan was developed based on these data (Figure 3).

Soil pH measured in the lab confirmed slightly alkaline values for most soil samples, as indicated by the USDA WSS database, but variations were observed within specific soil types. Overall, soil pH ranged between 7.15 and 8.39. Site averages ranged from 7.92 for soils of the Elkhills–Welport association to 7.61 for soils associated with the Littlesignal–Cochora complex. About 27% of the soil samples showed a pH of 8 and higher, but high pH values were not restricted to a particular soil type.

Similar variability was observed for soil electrical conductivity. Soil electrical conductivity averages ranged from 0.92 mS/cm in soils belonging to the Xerorthents–Badlands association to 0.36 mS/cm for soils of the Littlesignal–Cochora complex. High values of >1 were often observed (26%), independent of soil type. Overall, values ranged from as low as 0.1 to as high as 4.3 dS/cm.

### 3.4. Detection of Coccidioides

DNA was extracted successfully from all soil samples, and nested PCRs could be performed on all of them. The results to detect the pathogen differed between years. In 2018, 17 soil samples (41.14%) tested positive for *Coccidioides* compared to 5 samples (26.32%) in 2019, and 14 samples in 2021 (66.67%). Soil samples of the Beam–Panoza–Hillbrick association tested positive for the pathogen the most often. None of the samples collected from the Pyxo–Cochora association tested positive in 2019; however, a larger percentage of samples from this type of soil tested positive for the pathogen in 2018 and 2021. Of all the dominant soil types, the pathogen was the least often detected in soils that belonged to the Littlesignal–Cochora association, and more often in other dominant soils (Table 1).

With all soil samples combined, the pathogen was detected in 31.58% of them (n = 36). Out of these, *C. posadasii* was present in 83.33%, and *C. immitis* was detected in the remaining 16.67% of the samples.

**Figure 4:**
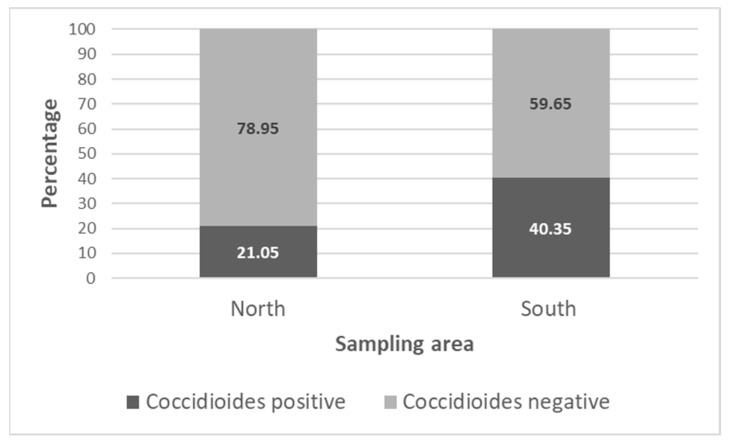
Percentage of positive and negative soil samples in the northern and southern sampling area for all soil samples collected in 2018, 2019, and 2021 (n = 114).

Overall, the pathogen was detected more frequently in soils collected in the southern sampling area (40.35%) where the environment appeared less degraded by cattle grazing, than in samples collected in the northern sampling area (21.05%) (Figure 4). In most cases, the diagnostic PCR indicated the presence of *C. posadasii*. However, *C. immitis* was detected in six soil samples. In 2018, this species was found in three soils of the southern sampling area (T4200-2, T4200-11, and T4500-7), and was not detected in the northern area. In 2019 and 2021, *C. immitis* was detected in the northern sampling area only (2019: T40-4200-8B; 2021: T40-4200-6 and T4300-1). *C. immitis* was detected in all dominant soil types: Xeric Torriorthents–Badlands complex (n = 1), the Pyxo–Cochora association (n = 1), the Elkhills–Welport association (n = 1) association, Littlesignal–Cochora association (n = 1), and the Beam–Panoza–Hillbrick association (n = 2), as was *C. posadasii,* which was detected in all sampling areas in all years (n = 34) in all dominant soil types.

**Figure 5:**
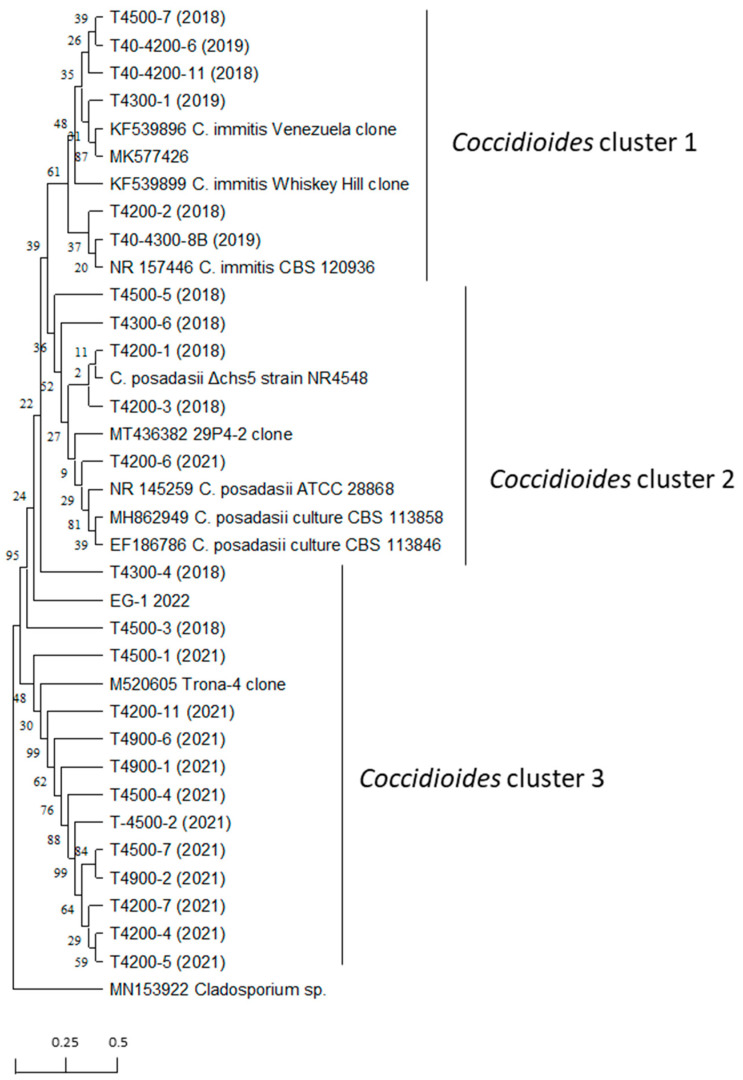
Phylogenetic tree of PCR amplicons obtained with primer pair EC3/EC100 together with close matches in the GenBank database (*Cladosporium* sp. was used as outgroup). The evolutionary history was inferred using the neighbor-joining method. The percentage of replicate trees in which the associated taxa clustered together in the bootstrap test (1000 replicates) is shown next to the branches. The evolutionary distances were computed using the maximum composite likelihood method and are in the units of the number of base substitutions per site. Evolutionary analyses were conducted in MEGA11. All ambiguous positions were removed for each sequence pair (pairwise deletion option). There were a total of 577 positions in the final dataset.

Examples of the PCR results obtained with the diagnostic primer pair EC3/EC100 are shown in Supplementary Figure S2. The closest matches to the PCR amplicons obtained with the *Coccidioides*-specific primer pairs EC3/EC100 were *C. immitis* (GenBank Accession # MK577426) and *C. posadasii* (GenBank Accession # MT436388). The positive amplicons were between 99 and 100% related to these database entries. A representative subset of all the sequenced amplicons was submitted to GenBank (OP902901-OP902924). A phylogenetic tree of the representative amplicons together with reliable entries from the GenBank database was conducted which separated the ~570 bp amplicons into three clusters that differed only slightly (Figure 5).

**Figure 6:**
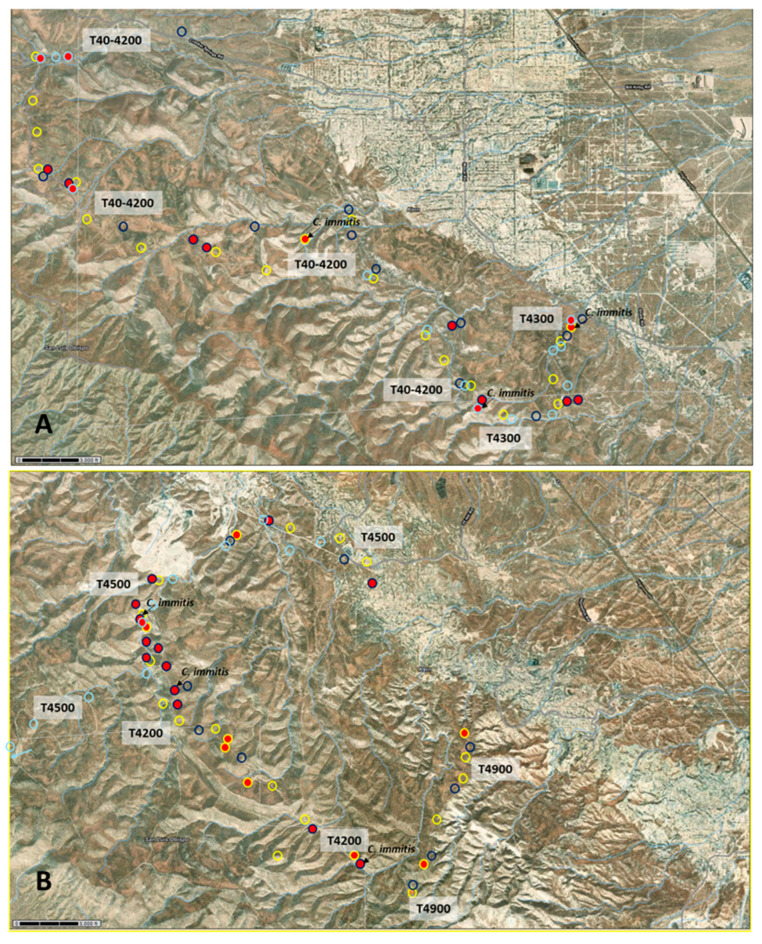
Locations of sampling sites along trails. (**A**) T4500, T4200, and T4900 in the southern sampling area. (**B**) Trails T40-4200 and T4300 in the northern sampling area (2018: dark blue circles, 2019: light blue circles, 2021: yellow circles). Sites where *Coccidioides* spp. were detected are indicated by circles filled in red. In addition, sites where *C. immitis* was detected are labeled.

Figure 6A,B shows all the sampling sites in the SRMA with those testing positive for the *Coccidioides* highlighted. The southern sampling area shows a cluster of positive sites in the northwest at the end of trail T4200 and at the beginning of trail T4500, but scattered positive sites were found along all trails. In the northern sampling area, *Coccidioides* were detected more randomly along all trails.

### 3.5. Environmental Parameters and Coccidioides

We investigated whether samples collected from different soil types in the Temblor SRMA display significant differences in pH and EC, and whether any such differences might be associated with *Coccidioides* presence. These analyses excluded those soil types for which <5 pH and EC measurements were obtained and included samples collected from soils of the Beam–Panoza–Hillbrick-(n = 42), Pyxo–Cochora-(n = 27), Elkhills–Welport-(n = 13), Littlesignal–Cochora-(n = 10), and Xeric–Torriorthents–Badlands (n = 8) associations.

A visual analysis of the distribution graphs and quartile-quartile (QQ) plots of the residuals of the pH data revealed that error was approximately normally distributed for these data. A two-factor ANOVA with type III sum of squares on pH with year and soil type as factors was run to determine if samples could be pooled across soil types and/or years. This analysis found no interaction effect (F_7,86_ = 0.45, *p* > 0.8) and no effect of soil type (F_4,86_ = 1.19, *p* > 0.3), but a significant effect of year was discovered (F_2,86_ = 5.67, *p* < 0.005). This result justifies pooling samples across soil types, but not across years. Therefore, to determine if pH was associated with *Coccidioides* presence, individual one-factor ANOVA were run for each year, with sites pooled. The results of these analyses are presented in the Supplementary Materials, Table S4. There was no significant difference in either the 2018 or 2019 samples, but in 2021, soils with higher pH values were less likely to contain *Coccidioides* than soils with lower pH (F_1,39_ = 11.0, *p* < 0.005).

A visual analysis of electrical conductivity (EC) residuals (distribution and QQ-plots) showed that the error for the EC data was not normally distributed. Natural log transformation of the EC data successfully normalized the residuals, and all subsequent statistical tests use the transformed data. A two-factor ANOVA with type III sum of squares on EC with year and soil type as factors was run to determine if samples could be pooled across soil types and/or years. This analysis found no interaction effect (F_7,86_ = 0.59, *p* > 0.7) and no effect of soil type (F_4,86_ = 2.25, *p* > 0.05), but revealed a significant effect of year (F_2,86_ = 14.9, *p* < 0.005). Again, this result justifies pooling samples across soil types, but not across years. To determine if EC was associated with *Coccidioides* presence, individual one-factor ANOVA were run for each year, with sites pooled. The results of these analyses are presented in the Supplementary Materials, Table S4. There was no significant difference in either the 2019 or 2021 samples, but in 2018, soils with higher EC values were more likely to contain *Coccidioides* than soils with lower EC (F_1,34_ = 5.84, *p* < 0.05).

Of all the five dominant soil types sampled, two stood out for having more than 35% of their individual soil samples testing positive for the pathogen. These soils belonged to the Beam–Panoza–Hillbrick complex and the Pyxo–Cochora association. Soils of the Littlesignal–Cochora association were least likely to test positive for *Coccidioides*, compared to other dominant soil types, with only ~18% of the soil samples testing positive. Only soil types for which over seven individual soil samples were collected were included in this evaluation. Of those soil types that were less common and where few soil samples were collected, only one included a *Coccidioides* positive soil sample (Pyxo–Kimberlina–Cochora association, map unit # 472, northern sampling area). The few samples collected from soils belonging to the Reward–Hillbrick association, Guijarral gravelly sandy loam, Padres sandy loam, and Xerorthents–Badlands complex did not include a *Coccidioides* positive soil sample. Photos of sites where *Coccidioides* were detected are documented in Supplementary Figure S3.

**Figure 7:**
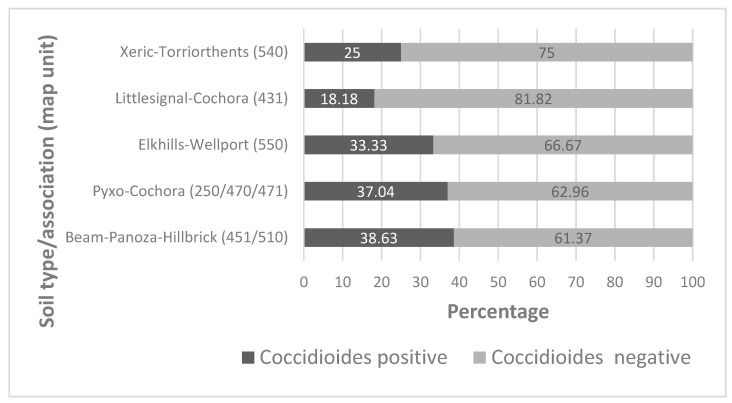
Percentage of *Coccidioides*-positive and -negative soil samples for different soil types collected between 2018 and 2021 (Xeric–Torriorthents: n = 8, Littlesignal–Cochora: n = 11, Elkills–Wellport: n = 12, Pyxo–Cochora: n = 27, Beam–Panoza–Hillbrick: n = 44).

Figure 7 provides the percentage of positive samples in each soil type, pooled over the years of the study. There is no difference in the frequency of *Coccidioides* in the five soil types (Χ^2^_4_ = 4.46, *p* > 0.3) represented.

**Figure 8:**
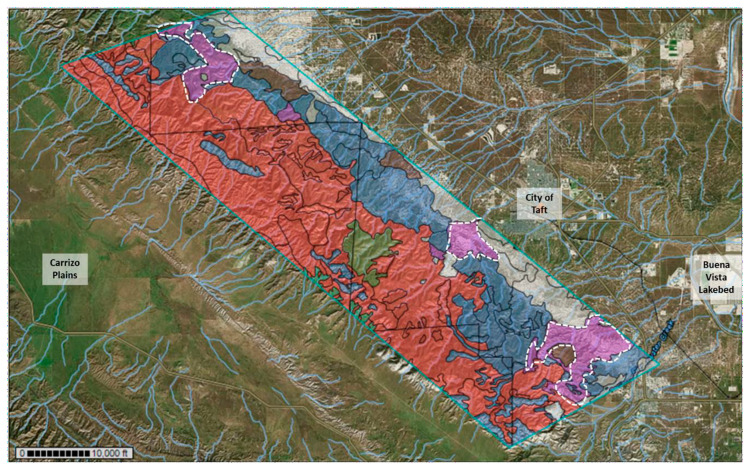
Map of the Temblor SRMA showing the extent of different soil types: Beam–Panoza–Hillbrick complex (red), Pyxo–Cochora–Badlands association (dark blue), Xeric Torriorthents– Badlands (light blue), Littlesignal–Cochora association (magenta), Reward channery loam (kaki), Elkhills–Welport association (grey), Elkhills–Pyxo association (brown), Padres sandy loam (green). Soils of the less abundant Xerorthents–Badlands association are not seen in this overview. The white dashed lines circle soils of the Littlesignal–Cochora association, which of all dominant soil types was the one with the least amount of *Coccidioides*-positive soil samples.

Based on the results of this study, considering the dominant soil types in the Temblor SRMA, the areas with the lowest exposure risk to *Coccidioides* appear to be soils of the Littlesignal–Cochora association. Two large patches of this type of soil can be found in the southern sampling area and an additional one can be found in the northern sampling area (Figure 8).

## 4. Discussion

Overall, the distribution of *Coccidioides* appeared scattered in the Temblor SRMA. The presence of the pathogen was confirmed for all dominant soil types in the area. The predominant soil type in the SRMA belongs to soils of the Beam–Panoza–Hillbrick association, followed by soils of the Pyxo–Cochora association, with more than 35% of soil samples testing positive for the pathogen in samples collected from each of these soils. Thus, disturbing any of these soil types will result in a high pathogen exposure risk. The analysis of soils of the Elkhills–Welport association, located predominantly in the eastern foothills of the SRMA, resulted in ~31% positive samples, indicating a high risk of potential pathogen exposure when disturbing this type of soil. In contrast, samples from the Littlesignal–Cochora complex, another dominant soil type in the SRMA, were the least likely to contain the pathogen, although 18% of soil samples of this type tested positive for the pathogen, as well. However, this difference was not found to be significantly different with the limited dataset available. Additional samples from soils of the Littlesignal–Cochora association could be collected and analyzed to confirm this observation. Based on these results, one may conclude that the exposure risk to *Coccidioides* arthroconidia is high when disturbing any of these dominant soil types.

The detection of the pathogen did not appear to be strongly correlated with soil pH or EC when samples from each year were combined. However, when results from each year were investigated individually, we detected that for the year 2021, soil pH was significantly lower for *Coccidioides*-positive samples, and in 2018, the EC of soil samples in which the pathogen was detected was significantly elevated. Not finding these correlations for the other years is most likely explained by yearly variation in weather and other environmental factors, but could be simply due to random sampling error, given the small sample sizes in some sites and years. Soil pH and EC are known to have a strong effect on microbial community structure and are influenced by weather events, plant exudates and microbial activities in the soil [26], and *Coccidioides* are known to be adapted to alkaline soil conditions, whereas most other soil fungi prefer slightly acidic or neutral conditions [27,28,29]. This makes sense, but a long-term study with detailed weather data would be needed to verify this observation. Statistical modelling has been applied to investigate the changes in Valley fever incidence over time with changes in weather data. Only a weak correlation between weather data could be documented for Kern County in California [30], compared to areas in Arizona where a stronger correlation was found [31] using a different statistical model.

Exposure risk may fluctuate with changes in the soil microclimate, both seasonally and with longer term changes in climate. Environmental soil parameter analyses revealed that the Temblor Mountain SRMA, with its elevated soil electrical conductivity and a pH that is mildly alkaline for most of the soil samples investigated, is a suitable habitat for *Coccidioides* and explains the large percentage of *Coccidioides*-positive soil samples. However, not all samples that fit into this description tested positive for the pathogen, as other factors including biological factors, such as plants and competing microbes, might play a role in determining the success of the pathogen’s establishment. From a microbe’s perspective, a soil environment that appears uniform to a human investigator, has many microhabitats that are characterized by different parameters which are influenced by temporal and seasonal changes. These differences may be enough to favor the growth of one species, or a guild of species, and inhibit others in different microhabitats of the soil matrix. This may explain the observed scattered distribution of *Coccidioides* in the soil environment, and thus account for the variation in detection frequency at sampling spots along certain trails. The scattered distribution of *Coccidioides* and the randomness of its presence in soil and dust samples has been documented in the literature [32,33,34].

We have shown that two key environmental parameters, soil pH and electrical conductivity, are elevated enough in the Temblor Mountain SRMA to support the growth of *Coccidioides* throughout the area. The Temblor Mountains were also predicted to be a suitable habitat for the pathogen based on environmental parameters including precipitation, soil type, pH, and EC [35]. By detecting the pathogen in each sampling season in deeper soils, we can conclude it is well established in that area and not simply blown in from nearby established populations. An RNA-based approach to confirm that the pathogen is actively metabolizing at least at some time during the year in the soil could be the focus of future studies. Three larger areas were identified where soils of the Littlesignal–Cochora association are prevalent, which had the least amount of *Coccidioides*-positive soil samples (~18%). These areas pose the lowest risk of *Coccidioides* exposure for recreationists in the SRMA (Figure 8). However, to strengthen the statistical support for this statement, additional soil samples should be investigated.

Overall, the pathogen appears to be more prevalent in the southern sampling area which is less affected by cattle grazing and has a more natural landscape with many California native herbs and small bushes. This observation confirms the results from other studies in the Central Valley and the Mojave Desert which documented that *Coccidioides* thrive in more natural environments compared to established cattle ranches, agricultural fields or orchards. The reasons for this observation are debated, but it is assumed that due to the application of fertilizer and the reduced abundance of rodents, the soil environment becomes more favorable for those species that degrade plant-derived matter and is more disadvantageous for keratinophiles [14,36,37].

Every year, the Temblor SRMA attracts many hikers, plant enthusiasts, mountain bikers, and motorized off-road vehicle bikers, who are mostly unaware of the risk of contracting a potentially serious disease. Visitors who are enjoying the wildflower blooms in the spring or are observing birds during the nesting season earlier in the year, are at a lower risk of becoming exposed to high loads of airborne *Coccidioides* arthroconidia, because the soil is still moist and fugitive dust events are rare. In contrast, visitors in the dry season, independently of what their recreational activity is, may become exposed to at least some dust, therefore experiencing a higher risk of pathogen exposure. The highest risk, however, is experienced by those recreationists who disturb the soil during the dry season, such as off-road vehicle drivers and riders. Of all recreational activities, off-road-vehicle recreation creates the most soil disturbance, destroys the vegetation coverage, and creates significant dust plumes in areas with fine particulate soils that can be transported great distances during dust storms [38,39]. Once disturbed, soil remains a potential source of fugitive dust, and thus becomes a potential health hazard.

Information obtained from several public agencies, such as the California Department of Public Health (CDPH), the Centers for Disease Control and Prevention (CDC), Los Angeles County Public Health, as well as information from selected peer-reviewed literature, and the authors’ experience based on years of field work, were used to compile a list of recommendations and best management practices to prevent Valley fever for recreationists, as well as BLM employers and field workers on BLM land in the endemic area of *Coccidioides* spp., and this might be useful for anyone who is working or engaging in recreation in the endemic area of the pathogen. This information can be used for educational purposes for recipients without a scientific background and includes some basic facts on *Coccidioides* and *coccidioidomycosis,* for example, about the ecology of this fungus, signs and symptoms and treatment of the disease, and actions to preventing the misdiagnosis and dissemination of Valley fever. Furthermore, recommendations on delineating hot spots of the pathogen in its endemic area, predominantly in known hot spots of *Coccidioides*, as well as information about employer’s duties to reduce the risk of pathogen exposure for field workers, including sustainable management practices in the field were compiled, including original references and links to websites (Supplementary Material: Prevention of Valley Fever—Best Management Practices).

In conclusion, the Temblor Mountain SRMA is a favorable habitat for *Coccidioides*, and therefore, the pathogen exposure risk for visitors and workers can be determined as very high. Even though an alignment of all PCR amplicons obtained with the diagnostic primer pair EC3/EC100 revealed slight genetic differences among them, the short branches displayed in the phylogenetic tree that was compiled did not allow us to conclude without doubt that both species of *Coccidioides* are present in the soils of the Temblor SRMA. *C. posadasii* and *C. immitis* have been detected in some clinical samples outside their known endemic areas; however, travel history of these patients could not be verified without doubt [40,41,42]. Although *C. immitis* is traditionally thought to be the only species of *Coccidioides* in the Central Valley of California, this could change in the future with changing climate conditions. Santa Ana winds and increased soil disturbance in the Mojave Desert may allow increased fugitive dust emissions and the transport of fungal spores with westerly winds, leading to an increased dispersal of *Coccidioides* arthroconidia to areas of Central California where it does not already occur. In fact, the transport of inland dust to coastal California areas and the Channel Islands has been documented [43,44,45], including documented Valley fever infections in marine mammals [46,47]. The potential expansion of *Coccidioides* with climate change is being discussed in the scientific community, and it seems reasonable to believe that a change to drier and hotter climates in non-traditional areas of *Coccidioides* may lead to an increase in disease incidence among humans [48,49].

## Figures and Tables

**Table 1:** Soil types in the Temblor SRMA that tested positive for *Coccidioides*. The table combines results for the southern and northern sampling areas for all three sampling events. The percentage of positive results for specific soil types is indicated. Soil types are color coded to match Figure 1.

Sampling, November 2018			
Soil Type and Soil Map Unit #	Number of Soil Samples n (North = 18, South = 23)	Detection of *Coccidioides* (n)	Detection of *Coccidioides* (%)
Elkhills-Welport association (550)	1 (north), 3 (south)	0 (north), 1 (south)	25.00
Pyxo-Cochora association (470, 471)	3 (north), 6 (south)	1 (north), 4 (south)	55.55
Beam-Panoza-Hillbrick association (510, 221, 223, 227, 451)	8 (north), 7 (south)	3 (north), 4 (south)	46.67
Littlesignal-Cochora association (431)	2 (north), 3 (south)	1 (north), 0 (south)	20.00
Elkhills-Pyxo association (440)	1 (north), 0 (south)	0 (north), 0 (south)	0
Pyxo-Kimberlina-Cochora association (472)	1 (north), 0 (south)	1 (north), 0 (south)	100.00
Xeric-Torrirothents-Badlands complex (249, 540)	1 (north), 4 (south)	0 (north), 2 (south)	40.00
Reward-Hillbrick association (580)	1 (north), 0 (south)	0 (north), 0 (south)	0
**Sampling, June 2019**			
**Soil Type and Soil Map Unit #**	**Number of Soil Samples n (North = 19, South = 10)**	**Detection of *Coccidioides* (n)**	**Detection of *Coccidioides* (%)**
Elkhills-Welport association (550)	4 (north), 0 (south)	1 (north), 0 (south)	25.00
Elkhills-Pyxo association (440)	1 (north), 0 (south)	0 (north), 0 (south)	0
Pyxo-Cochora association (470, 471)	1 (north), 2 (south)	0 (north), 0 (south)	0
Beam-Panoza-Hillbrick association (510, 221, 223, 227, 451)	10 (north), 4 (south)	3 (north), 1 (south)	28.57
Littlesignal-Cochora association (431)	0 (north), 2 (south)	0 (north), 0 (south)	0
Pyxo-Kimberlina-Cochora association (472)	0 (north), 0 (south)	not re-sampled	0
Xeric-Torrirothents-Badlands complex (249, 540)	0 (north), 0 (south)	not re-sampled	0
Reward-Hillbrick-association (580)	0 (north), 0 (south)	not re-sampled	0
Guijarral gravelly sandy loam (193)	0 (north), 1 (south)	0 (north), 0 (south)	0
Xerorthents-Badlands complex (219)	3 (north), 0 (south)	0 (north), 0 (south)	0
Padres sandy loam (490)	0 (north), 1 (south)	0 (north), 0 (south)	0
**Sampling March 2021**			
**Soil Type and Soil Map Unit #**	**Number of Soil Samples n (North = 20, South = 24)**	**Detection of *Coccidioides* (n)**	**Detection of *Coccidioides* (%)**
Elkhills-Welport association (550)	3 (north), 2 (south)	0 (north), 2 (south)	40.00
Pyxo-Cochora association (470, 471)	5 (north), 9 (south)	0 (north), 5 (south)	35.71
Beam-Panoza-Hillbrick association (510, 221, 223, 227, 451)	5 (north), 10 (south)	0 (north), 5 (south)	40.00
Littlesignal-Cochora association (431)	3 (north), 1 (south)	0 (north), 0 (south)	25.00
Elkhills-Pyxo association (440)	1 (north), 0 (south)	0 (north), 0 (south)	0
Pyxo-Kimberlina-Cochora association (472)	1 (north), 2 (south)	0 (north), 0 (south)	0
Xeric-Torrirothents-Badlands complex (249, 540)	1 (north), 0 (south)	0 (north), 0 (south)	0
Polonio clay loam (170)	1 (north), 0 (south)	0 (north), 0 (south)	0
Xerorthents-Badlands complex (219)	0 (north), 0 (south)	0 (north), 0 (south)	0

## Data Availability

DNA sequence data was deposited in GenBank, accession numbers OP902901-OP902924.

## Supplementary Materials

The following supporting information can be downloaded at: https://www.mdpi.com/article/10.3390/microorganisms11020518/s1, Figure S1: Landscape overviews along trails in the southern and northern sampling areas of the Temblor SRMA, March 2021. Locations of individual sampling sites are indicated on the bottom of each picture. Cattle can be seen grazing in the northern sampling area (bottom row, very right). Figure S2: Percentage of biological samples testing positive for *Coccidioides*. (Biological Soil Crust [BSC]: n = 3; snake feces: n = 2; cattle patties: n = 5; rodent pellets: n = 11; mammalian predator feces: n = 10). Figure S3: Photos of sampling sites that tested positive for *Coccidioides* spp. Individual sampling sites are indicated in each picture (bottom).; Table S1: Coordinates, elevation, and brief site description of all soil samples collected in the northern and southern sampling area of the Temblor SRMA (11/02/2018). Soil samples in which *Coccidioides* was detected are in bold. Table S2: Coordinates, elevation, and brief site description of all soil samples collected in the southern and northern sampling area of the Temblor SRMA (06/17/2019). Soil samples in which *Coccidioides* was detected are in bold. Supplementary Table S3: Coordinates, elevation, and brief site description of all soil samples collected in the southern and northern sampling area of the Temblor SRMA (03/21/2021). Soil samples in which *Coccidioides* were detected are indicated in bold (clustering with *C. posadasii*), or in yellow (clustering with sequences from *C. immitis*) (based on sequence clustering in Figure 5). Supplementary Table S4: Comparing electrical conductivity (EC) values for different soil types. Tukey test results of re-transformed data. Supplementary Table S5: Descriptive statistics with results of one-way ANOVAs by year for pH and EC (mS/cm) for each sampling year for presence/absence of the pathogen (** significant difference) [50,51,52,53,54,55,56].
