# Supplementary material for: Risk of Exposure to Coccidioides spp. in the Temblor Special Recreation Management Area (SRMA), Kern County, CA
Source: Microorganisms. 2023 Feb 17;11(2):518. doi: 10.3390/microorganisms11020518 (PMC9964165; doi:10.3390/microorganisms11020518)
Supplement: Supplementary file 1 [file microorganisms-11-00518-s001.zip › microorganisms-2207442-supplementary.docx]

**Supplementary Material**

## Prevention of Valley Fever - Best Management Practices

1. **Delineate hot spots for *Coccidioides*** on BLM land.
   1. Investigate any recreational area where soil disturbance might occur during some time of the year due to natural causes or due to human activity for the presence of soil-borne pathogens, including *Coccidioides*, that can pose a substantial healthcare burden on individuals should they become infected.
   2. Prioritize soil analysis for the pathogen on BLM lands where significant soil disturbance may occur due to human activity and where humans or animals (primarily dogs) are known to have contracted the pathogen, as well as areas where large numbers of visitors are expected during the dry season of the year.
2. **Implement training about Valley fever** for BLM employees who are spending time in the field and for visitors to recreational areas that scientists have identified as *Coccidioides* hot spots. AB (Assembly Bill) 203 of 2019, introduced on May 1, 2020, is a recent California law that affects employers who operate in counties with high incidence of Valley fever. Training should take place annually about how to reduce the risk of Valley fever in the workplace. This mandate may be particularly relevant as well for managers of renewable energy projects, for example, in the San Joaquin Valley/Tulare Basin and in the West Mojave Desert. BLM personnel can be trained in seminars (see topics to be addressed below) given by a trained supervisor or by a consultant with experience in Valley fever. Training guides for California construction workers have been developed by the California Department of Public Health [50] and the County of Los Angeles [51].

Furthermore, a factsheet with information on the disease can be distributed to visitors. The goal is to minimize the risk of contracting Valley fever by being informed about how the disease is contracted and how disease symptoms typically manifest in a patient, making employees and visitors aware of the seriousness of this illness. The federal Center for Disease Control (CDC) has developed a factsheet to increase Valley fever awareness [52]. Topics to be covered in an educational session should include:

- 1. General information on the ecology of *Coccidioides* in California: Soil borne, spore-forming opportunistic pathogen that can cause coccidioidomycosis when inhaled and when established in lungs.
  2. Typical signs and symptoms of the disease, once contracted, especially, fever, night sweats, extreme fatigue, rash (erythema nodosum), dry cough, shortness of breath, and joint pain
  3. Information about incidence of Valley fever in California (or in specific counties) over time. Data are available on the websites of the CDC [53] and the California Department of Public Health [54]
  4. Information on estimated yearly burden to the health care system and treatment costs for Californians [7-10, main manuscript].
  5. Facts on currently available treatment options:
     1. No vaccine for humans is currently available.
     2. Commonly used azole drugs only inhibit the pathogen in the human body and have significant side effects for patients.
     3. Only a robust immune system can cure a patient.
     4. People should request a test for Valley fever from their health care provider when they feel sick several days to weeks after spending time outdoors in the endemic area of the pathogen. Note: A blood test for antibodies might be false negative when the disease is in its early stage. Therefore, a request for antigen testing is appropriate or re-testing for antibodies later is recommended.
  6. Prevention of exposure to the pathogen:
     1. It is impossible to reduce pathogen exposure risk to zero, but some recreational areas identified as hot spots of the pathogen bear a higher risk of people becoming infected when spending time in that area, especially when unprotected.
     2. Avoid unnecessary soil disturbance.
     3. Wear a dust mask and other personal protective equipment (PPE) when exposed to dust (N95 dust mask recommended by CDC).
     4. Wash clothing and equipment every day after use. Be aware that arthroconidia (spores) of the pathogen can be on your clothes and equipment.
     5. Restoring natural vegetation in areas where human activity or natural causes have created bare soil, where feasible.
     6. Consider groundcovers, such as gravel, or mulch on dirt parking lots where soil is disturbed.
     7. Contact recreation visitors about compliance with health guidelines for Valley fever and about health risks resulting from non-compliance.
     8. Prepare information leaflets as handouts for visitors.
     9. Ensure that BLM employers and employees are aware of recommendations and consider seriously personal consequences of carelessness. Consider fines for violations (e.g., destroying a fence, disturbing restored areas, damaging endangered plants, harassing wildlife, illegal shooting, illegal dumping of trash etc.) and enforce them.
     10. Restrict certain types of recreational activities in identified hot spots of the pathogen, especially off-road biking.
     11. Provide worker training in Valley fever prevention and management steps to avoid soil disturbances in areas with high incidence of Valley fever. For instructional materials and media see [55].
  7. Prevent severe cases of pathogen infection:
     1. Be aware that *C. immitis* and *C. posadasii* can spread to any organ if a person’s immune system fails to eliminate the pathogen. It spreads via the blood system and lymphatic system and can even cause meningitis.
     2. Be educated on typical signs and symptoms of Valley fever [56].
     3. Visit a healthcare provider if you become sick.
     4. Be proactive and make clear to the health care provider that you contracted Valley fever. Do not wait for the health care provider to try out different antibacterial treatments first before an antifungal treatment when symptoms and signs are already severe. Delayed diagnosis of Valley fever has resulted in immense human suffering and increased treatment costs.

## Best Management Practices in the Field

- 1. Avoid Occupational Safety and Health Administration (OSHA) citations by educating employers and employees.
  2. Provide protective equipment to employees when work results in their exposure to dust.
     1. BLM employees involved in work that requires major soil disturbance (e.g., trail maintenance, restoration efforts) should undergo a Valley fever antibody test prior to work on site to determine whether they are at risk of contracting the disease or if they are immune.
     2. BLM employees with conditions that negatively impact immune system function, or their lungs are at a higher risk of contracting Valley fever when working in dusty environments. The following employee groups are at elevated risk: drug and alcohol addicts, diabetics, HIV positive people, people with chronic infectious diseases, asthmatics, people who take steroids regularly, chain smokers and vapers, and older employees.
     3. Short- and long-term dust mitigation must be considered to protect all people from contracting Valley fever.

Keeping the dust from becoming airborne by watering the ground is a short-term mitigation method but might trigger growth of *Coccidioides* spp. with increased soil moisture. A site that might have contained few spores of the pathogen might become a hot spot and poses a risk for visitors and nearby residents.

- - 1. A list of control measures, compiled by the California Department of Public Health (CDPH 2020).
    2. Speak to your field office safety officer when you see a lack of effective dust control mitigation methods (e.g., lack of personal protective equipment (PPE) and BLM-sponsored education about Valley fever.
    3. The Cal/OSHA Consultation Services Branch (https://www.dir.ca.gov/dosh/consultation.html) is available for workplace safety regarding Valley fever prevention at 1-800-963-9424 or email [infocons@dir.ca.gov](mailto:infocons@dir.ca.gov).

References

[50] Valley Fever, Tailgate Training Guide for California Construction Workers; California Department of Public Health (CDPH). 2020. Available online: https://ucanr.edu/sites/safety/files/333256.pdf (accessed on 11 November 2022).

[51] Acute Communicable Disease Control Program and Department of Environmental Health, Coccidioidomycosis (Valley Fever) Management Plan: Guidelines for Employers; County of Los Angeles Public Health. Published August 2019. Available online: [valleyfeverplan2019.pdf (lacounty.gov)](http://publichealth.lacounty.gov/acd/docs/valleyfeverplan2019.pdf) (accessed 11 November 2022).

[52] Be Aware of Valley Fever; Centers for Disease Control and Prevention (CDC), Mission and Community Service Groups. Published September 2019. Available online: <https://www.cdc.gov/fungal/diseases/coccidioidomycosis/pdf/be-aware-of-valley-fever-h.pdf> (accessed 11 November 2022).

[53] Valley Fever (Coccidioidomycosis) Statistics, CEDC 24/7: Saving Lives, Protecting People™, Centers for Disease Control and Prevention (CDC). 2022. Available online <https://www.cdc.gov/fungal/diseases/coccidioidomycosis/statistics.html> (accessed 11 November 2022).

[54] Valley Fever is on the Rise in California; California Department of Public Health (CDPH). 2022. Available online: [Coccidioidomycosis (Valley Fever) (ca.gov)](https://www.cdph.ca.gov/Programs/CID/DCDC/Pages/Coccidioidomycosis.aspx) (accessed on 11 November 2022).

[55] Dusty Work Increases Valley Fever; California Department of Public Health (CDPH), Occupational Health Branch. Published September 2022. Available online: [Work-Related Valley Fever (Coccidioidomycosis) (ca.gov)](https://www.cdph.ca.gov/Programs/CCDPHP/DEODC/OHB/Pages/Cocci.aspx) (accessed on 11 November 2022).

[56] Valley Fever, Symptoms; California Department of Public Health (CDPH). August 2022. Available online: [Valley Fever Symptoms (ca.gov)](https://www.cdph.ca.gov/Programs/CID/DCDC/Pages/ValleyFeverSymptoms.aspx) (accessed on 11 November 2022).

**
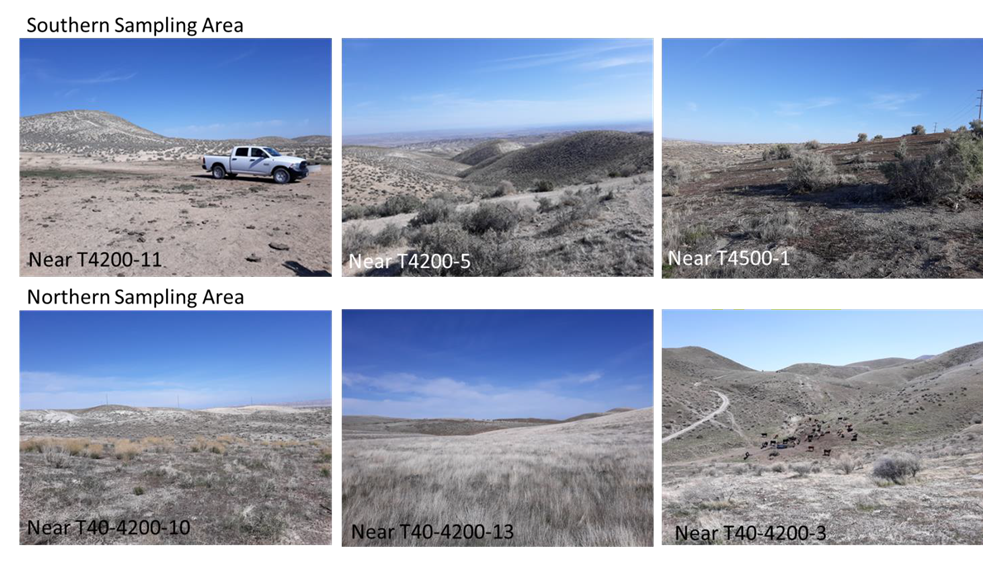
**

**Supplementary Figure S1.** Landscape overviews along trails in the southern and northern sampling areas of the Temblor SRMA, March 2021. Locations of individual sampling sites are indicated on the bottom of each picture. Cattle can be seen grazing in the northern sampling area (bottom row, very right).


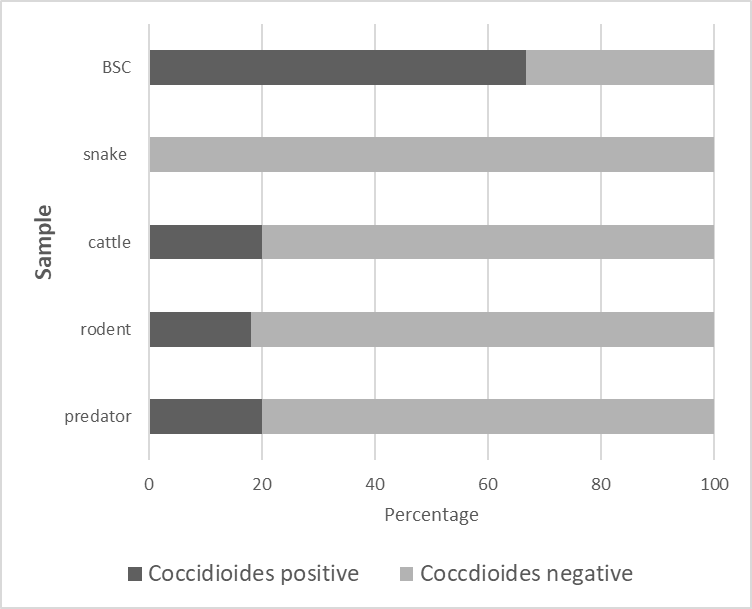


**Supplementary Figure S2.** Percentage of biological samples testing positive for *Coccidioides*. (Biological Soil Crust [BSC]: n=3; snake feces: n=2; cattle patties: n=5; rodent pellets: n=11; mammalian predator feces: n=10).


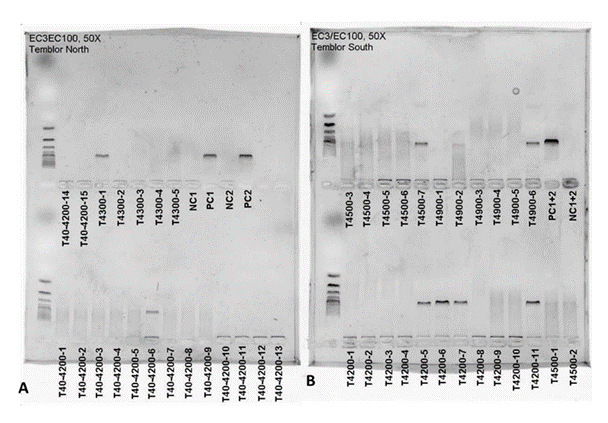


**Supplementary Figure S3.** PCRs obtained with the *Coccidioides* specific primer pair EC3/EC100 for samples collected in the northern **(A)** and the southern **(B)** Temblor SRMA in 2021 (positive and negative controls (PC, NC) were included; size of PCR amplicon: ~550 bp).


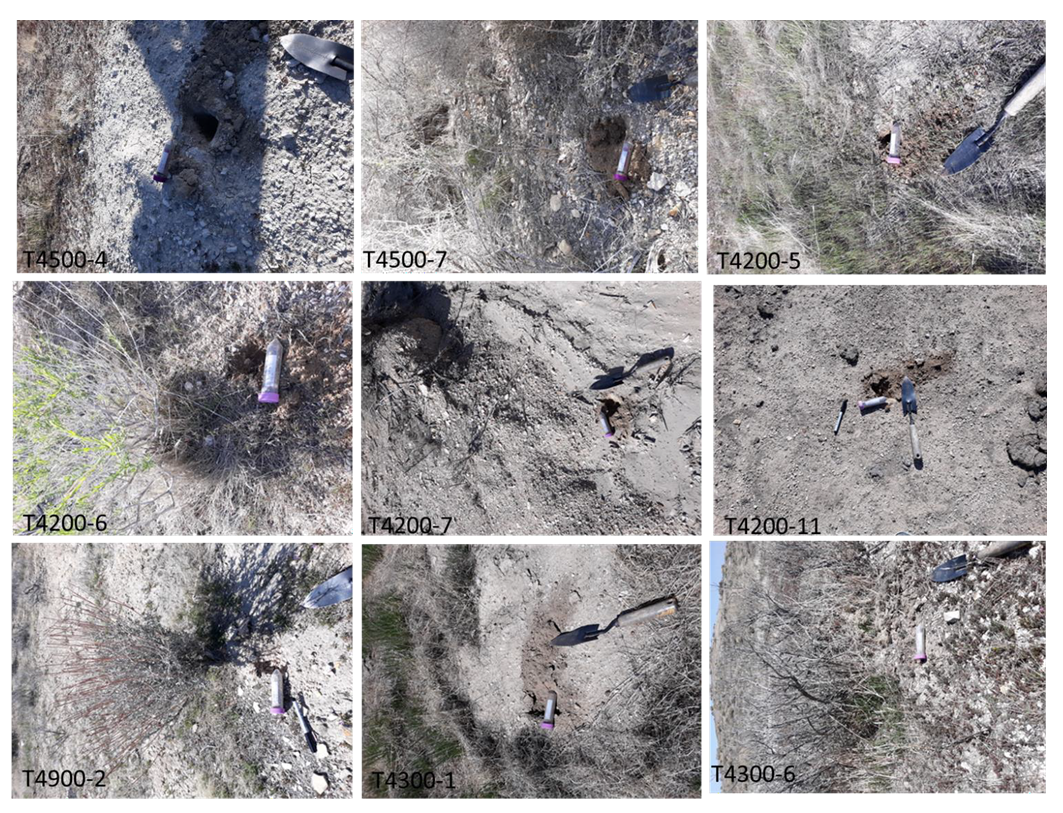


**Supplementary Figure S4.** Photos of sampling sites that tested positive for *Coccidioides* spp. Individual sampling sites are indicated in each picture (bottom).

**Supplementary Table S1.** Coordinates, elevation, and brief site description of all soil samples collected in the northern and southern sampling area of the Temblor SRMA (**11/02/2018**). Soil samples in which *Coccidioides* was detected are in bold.

| **Sampling site** | **Coordinates** | | **Elevation (ft)** | **Site description** |  |  |  |  |  |  |
| --- | --- | --- | --- | --- | --- | --- | --- | --- | --- | --- |
| **Northern sampling area** | | | | |  |  |  |  |  |  |
| T4300 (1) | 35° 11’ 29.56” N | 119° 34’ 43.18” W | 1,444 | low hills, near entrance, BSC |  |  |  |  |  |  |
| T4300 (2) | 35° 11’ 15.53” N | 119° 34’ 54.73” W | 1,517 | near Bladder Pod (*Peritoma arborea*) |  |  |  |  |  |  |
| **T4300 (3)** | 35° 10’ 39.88” N | 119° 35’ 54.61” W | 1,797 | near Saltbush (*A. spinifera*), compacted soil |  |  |  |  |  |  |
| **T4300 (4)** | 35° 10’ 34.92” N | 119° 35’ 19.05” W | 1,861 | rodent wall |  |  |  |  |  |  |
| T4300 (5) | 35° 10’ 41.68” N | 119° 35’ 04.15” W | 1,813 | eroded compacted soil |  |  |  |  |  |  |
| **T4300 (6)** | 35° 10’ 40.27” N | 119° 35’ 39.81” W | 1,984 | wall |  |  |  |  |  |  |
| 40-T4200 (1) | 35° 10’ 51.39” N | 119° 35’ 48.89” W | 2,106 | uphill, rodents, *A. spinifera* |  |  |  |  |  |  |
| 40-T4200 (2) | 35° 11’ 13.71” N | 119° 36’ 15.02” W | 1,902 | broken side of road |  |  |  |  |  |  |
| 40-T4200 (3) | 35° 11’ 36.95” N | 119° 36’ 35.96” W | 1,896 | loamy side of road |  |  |  |  |  |  |
| 40-T4200 (4) | 35° 11’ 50.06” N | 119° 36’ 49.51” W | 1,940 | ditch |  |  |  |  |  |  |
| 40-T4200 (5) | 35° 12’ 12.71” N | 119° 37’ 03.75” W | 1,811 | non-vegetated area |  |  |  |  |  |  |
| 40-T4200 (6) | 35° 12’ 13.97” N | 119° 38’ 00.61” W | 2,070 | grassy area |  |  |  |  |  |  |
| **40-T4200 (7)** | 35° 11’ 58.85” N | 119° 38’ 36.04” W | 2,486 | along roadside, hill |  |  |  |  |  |  |
| **40-T4200 (8)** | 35° 11’ 55.02” N | 119° 38’ 16.74” W | 2,374 | loamy side of road |  |  |  |  |  |  |
| 40-T4200 (9) | 35° 12’ 06.91” N | 119° 39’ 04.58” W | 2,300 | bottom of wash |  |  |  |  |  |  |
| **40-T4200 (10)** | 35° 12’ 23.07” N | 119° 39’ 39.79” W | 2,428 | caved in site, golden rod |  |  |  |  |  |  |
| 40-T4200 (11) | 35° 12’ 36.58” N | 119° 40’ 07.81” W | 2,470 | devastated by cows, grassy area, few golden rods (cattle poop samples) |  |  |  |  |  |  |
| 40-T4200 (12) | 35° 14’ 06.34” N | 119° 39’ 33.91” W | 2,226 | dense Saltbush area (*A. polycarpa*), compacted soil, way out, near farm |  |  |  |  |  |  |
| **Southern sampling area** | | | | |  |  |  |  |  |  |
| T4500 (1) | 35° 07’ 03.09” N | 119° 28’ 38.12” W | 1,622 | near oil rig |  |  |  |  |  |  |
| **T4500 (2)** | 35° 06’ 50.15” N | 119° 28’ 21.26” W | 1,607 | unmarked road, dry soil, white powdery, near salt bush |  |  |  |  |  |  |
| **T4500 (3)** | 35° 07’ 11.84” N | 119° 29’ 09.82” W | 1,525 | near rodent hole |  |  |  |  |  |  |
| T4500 (4) | 35° 07’ 02.60” N | 119° 29’ 38.33” W | 1,524 | near rodent hole, cattle guard |  |  |  |  |  |  |
| **T4500 (5)** | 35° 06’ 42.37” N | 119° 30’ 27.46” W | 1,764 | wash, samples taken at the side |  |  |  |  |  |  |
| T4500 (6) | 35° 06’ 27.35” N | 119° 30’ 37.24” W | 1,886 | along wash, hills |  |  |  |  |  |  |
| **T4500 (7)** | 35° 06’ 19.21” N | 119° 30’ 38.87” W | 1,948 | near wash, grasses, vertical rock/sediment layers |  |  |  |  |  |  |
| **T4200 (1)** | 35° 06’ 00.72” N | 119° 30’ 35.02” W | 2,040 | at intersection with T4200, potentially kit fox den entrance |  |  |  |  |  |  |
| **T4200 (2)** | 35° 05’ 39.58” N | 119° 30’ 27.60” W | 2,190 | along wash, compacted soil, center of wash |  |  |  |  |  |  |
| **T4200 (3)** | 35° 05’ 30.99” N | 119° 30’ 14.34” W | 2,367 | top of the hill, intersection of T3213, overview Taft |  |  |  |  |  |  |
| **T4200 (4)** | 35° 05’ 23.92” N | 119° 29’ 57.88” W | 2,277 | drainage, bottom of hill |  |  |  |  |  |  |
| T4200 (5) | 35° 05’ 21.11” N | 119° 29’ 48.31” W | 2,328 | top of hill, grasses, cow patty |  |  |  |  |  |  |
| **T4200 (6)** | 35° 05’ 22.60” N | 119° 29’ 47.16” W | 2,350 | other side of T4200-5, buckwheat |  |  |  |  |  |  |
| T4200 (7) | 35° 05’ 03.01” N | 119° 29’ 34.38” W | 2,258 | dirt-biking area, grey soils |  |  |  |  |  |  |
| T4200 (8) | 35° 05’ 02.32” N | 119° 29’ 20.10” W | 2,112 | valley, near water tank, *A. polycarpa*, fox mandible found, cow patty, grassy |  |  |  |  |  |  |
| T4200 (9) | 35° 04’ 19.69” N | 119° 29’ 07.27” W | 2,273 | hill near T3213 intersection to T4200A |  |  |  |  |  |  |
| **T4200 (10)** | 35° 04’ 41.02” N | 119° 28’ 59.49” W | 2,167 | eroded hill |  |  |  |  |  |  |
| **T4200 (11)** | 35° 04’ 24.14” N | 119° 28’ 29.64” W | 2,153 | mound, kangaroo rat and kit fox dens (fecal pellets collected) |  |  |  |  |  |  |
| T4900 (1) | 35° 04’ 05.24” N | 119° 27’ 53.68” W | 2,131 | near gate, Biological Soil Crusts (BSCs) |  |  |  |  |  |  |
| T4900 (2) | 35° 04’ 17.63” N | 119° 27’ 45.69” W | 2,116 | white soil, compacted, BSC (BSC collected) |  |  |  |  |  |  |
| T4900 (3) | 35° 04’ 44.06” N | 119° 27’ 36.01” W | 1,976 | no description |  |  |  |  |  |  |
| T4900 (4) | 35° 05’ 02.91” N | 119° 27’ 21.63” W | 1,826 | grassy area, mostly *Bromus* sp. |  |  |  |  |  |  |

**Supplementary Table S2.** Coordinates, elevation, and brief site description of all soil samples collected in the southern and northern sampling area of the Temblor SRMA **(06/17/2019)**. Soil samples in which *Coccidioides* was detected are in bold.

| **Sampling site** | **Coordinates** | | **Elevation (ft)** | **Site description** |
| --- | --- | --- | --- | --- |
| **Southern sampling area** | | | | |
| T4500-1 | 35°06’ 59.85” N | 119° 28’ 49.55” W | 1611 | near oilrig, rocky, organic matter, surface sample |
| T4500-2 | 35°06’ 56.21” N | 119° 29’ 08.87” W | 1561 | unmarked road, dry soil, white, powdery, near salt bush |
| T4500-3 | 35°07’ 11.37” N | 119° 29’ 23.90” W | 1548 | near rodent hole, eroded soil |
| T4500-4 | 35°07’ 00.42” N | 119° 29’ 47.96” W | 1708 | near cattle guard, rodent hole, dried annuals |
| T4500-5 | 35°06’ 43.06” N | 119° 30’ 18.23” W | 1705 | across kitty litter harvest, side of road, rodent holes, dusty soil |
| T4500-6 | 35°06’ 27.79” N | 119° 30’ 31.70” W | 1705 | near river wash, rodent holes, unvegetated |
| **T4500-7** | 35°06’ 10.85” N | 119° 30’ 40.78” W | 1951 | in eroded small ravine, washed out space, loose soil |
| T4500-8 | 35°05’ 55.82” N | 119° 30’ 46.28” W | 2124 | side of dry creek bed, rodent holes |
| T4500-9 | 35°05’ 41.41” N | 119° 31’ 13.33” W | 2570 | no description |
| T4500-10 | 35°05’ 26.82” N | 119° 31’ 45.05” W | 2998 | on side of road, dried annuals |
| T4500-11 | 35°05’ 12.69” N | 119° 32’ 09.27” W | 3406 | top of AT&T hill, grainy, hard soils |
| **Northern sampling area** | | | | |
| **T4300-1** | 35°11’ 21.61” N | 119° 34’ 45.40” W | 1534 | whitish soil, on side of road (no photo) |
| T4300-2 | 35°11’ 13.86” N | 119° 34’ 51.55” W | 1550 | on side of road, dried annuals |
| T4300-3A | 35°11’ 12.14” N | 119° 34’ 55.09” W | 1539 | ant nest (*Pogonomyrmex* sp.) |
| T40-4300-4A | 35°10’ 49.43” N | 119° 34’ 47.28” W | 1692 | soil near side of road, rocky, dead annuals |
| T40-4300-6A | 35°10’ 43.43” N | 119° 34’ 52.15” W | 1755 | hard soil |
| T40-4300-7 | 35°10’ 36.02” N | 119° 35’ 30.39” W | 1923 | in wash near salt bush, grey soil |
| T40-4300-7C | 35°10’ 36.02” N | 119° 35’ 30.39” W | 1923 | soil near dead salt bush, dead fiddle neck annuals (no photo) |
| T40-4300-7D | 35°10’ 36.02” N | 119° 35’ 30.39” W | 1923 | soil near rodent hole, dead annuals |
| **T40-4300-8** | 35°10’ 38.99” N | 119° 35’ 45.96” W | 2113 | powdery soil, like T40-4300-8b (no photo) |
| T40-4300-8B | 35°10’ 38.99” N | 119° 35’ 45.96” W | 2113 | whitish soil, near dried *Bromus* grass |
| T40-4200-1 | 35°10’ 51.98” N | 119° 35’ 52.94” W | 1991 | beside road, rodent holes, eroded soil |
| T40-4200-3 | 35°11’ 17.94” N | 119° 36’ 21.44” W | 1850 | rocky soil near ditch (no photo) |
| T40-4200-4A | 35°11’ 48.84” N | 119° 36’ 49.50” W | 1925 | side of road, rocky soil |
| T40-4200-4B | 35°11’ 48.84” N | 119° 36’ 49.50” W | 1925 | side of road, at rodent hole entrance |
| T40-4200-5 | 35°12’ 18.07” N | 119° 37’ 08.00” W | 1805 | side of road, loose soil (no photo) |
| T40-4200-6 | 35°12’ 39.60” N | 119° 40’ 04.76” W | 2445 | in small wash, rocky (no photo) |
| T40-4200-7 | 35°13’ 44.60” N | 119° 40’ 12.38” W | 2261 | grassy area, dead annuals, cow pasture |
| **T40-4200-8** | 35°13’ 43.99” N | 119° 40’ 28.56” W | 2302 | loose soil, on road near rodent hole, surface sample |
| **T40-4200-9** | 35°13’ 43.63” N | 119° 40’ 12.40” W | 2264 | on road, near rodent hole, surface sample |

**Supplementary Table S3.** Coordinates, elevation, and brief site description of all soil samples collected in the southern and northern sampling area of the Temblor SRMA **(03/21/2021)**. Soil samples in which *Coccidioides* were detected are indicated in bold (clustering with *C. posadasii*), or in yellow (clustering with sequences from *C. immitis*) (based on sequence clustering in Figure 5).

| **Site** | **Coordinates** | **Elevation (ft)** | **Site description** |
| --- | --- | --- | --- |
| **Southern area** |  |  |  |
| **T-4500-1** | 35° 07’ 04.44” N 119° 28’ 42.24” W | 1,614 | mix of brown and tan, sandy, little moisture, and organic matter |
| **T-4500-2** | 35° 06’ 49.68” N 119° 28’ 21.72” W | 1,680 | mix of brown and tan, sandy, little moisture, and organic matter |
| T-4500-3 | 35° 07’ 05.88” N 119° 29’ 08.52” W | 1,483 | mix of brown and tan, fine particulate, little moisture, and organic matter |
| **T-4500-4** | 35° 07’ 04.80” N 119° 29’ 43.80” W | 1,545 | mix of brown and tan, fine particulate, little moisture, and organic matter |
| T-4500-5 | 35° 06’ 41.40” N 119° 30’ 24.84” W | 1,732 | mix of brown and tan, gravelly, little moisture, and organic matter |
| T-4500-6 | 35° 06’ 26.28” N 119° 30’ 34.56” W | 1,847 | tan, fine particulate, dry, little organic matter |
| **T-4500-7** | 35° 06’ 15.12” N 119° 30’ 35.28” W | 1,893 | mix of brown and tan, sandy, little moisture, and organic matter |
| T-4200-1 | 35° 06’ 00.72” N 119° 30’ 36.00” W | 2,031 | brown, fine particulate, little moisture, and organic matter |
| T-4200-2 | 35° 05’ 40.92” N 119° 30’ 27.72” W | 2,162 | brown, little moisture with lots of organic matter |
| T-4200-3 | 35° 05’ 28.32” N 119° 30’ 17.64” W | 2,425 | light brown, dry, sandy, little organic matter |
| T-4200-4 | 35° 05’ 29.76” N 119° 30’ 03.60” W | 2,346 | brown, sandy, gravel, little moisture, and organic matter |
| **T-4200-5** | 35° 05’ 19.68” N 119° 29’ 54.96” W | 2,280 | brown, fine particulate, little moisture, and organic matter |
| **T-4200-6** | 35° 05’ 19.68” N 119° 29’ 54.96” W | 2,280 | mix of brown and tan, fine particulate, little moisture, and organic matter |
| **T-4200-7** | 35° 05’ 01.68” N 119° 29’ 35.52” W | 2,283 | brown, fine particulate, little moisture, and organic matter |
| T-4200-8 | 35° 04’ 58.44” N 119° 29’ 21.12” W | 2,156 | dark brown, little moisture and organic matter |
| T-4200-9 | 35° 04’ 30.00” N 119° 29’ 26.16” W | 2,484 | brown, fine particulate, little moisture, and organic matter |
| T-4200-10 | 35° 04’ 40.44” N 119° 29’ 01.32” W | 2,165 | tan, fine particulate, little moisture, and organic matter |
| **T-4200-11** | 35° 04’ 24.14” N 119° 28’ 29.64” W | 2,182 | brown, fine particulate, little moisture, and organic matter |
| T-4900-1 | 35° 04’ 05.24” N 119° 27’ 53.68” W | 2,116 | tan, fine particulate, little moisture, and organic matter |
| **T-4900-2** | 35° 04’ 17.63” N 119° 27’ 45.69” W | 2,129 | light tan, chipped, little moisture and organic matter |
| T-4900-3 | 35° 04’ 44.06” N 119° 27’ 36.01” W | 1,913 | fine particulate, dry, brown, little moisture, and organic matter |
| T-4900-4 | 35° 04’ 21.36” N 119° 28’ 28.56” W | 1,814 | brown, little moisture and organic matter |
| T-4900-5 | 35° 05’ 12.12” N 119° 27’ 19.08” W | 1,765 | mix of brown and tan, little moisture, and organic matter |
| **T-4900-6** | 35° 05’ 23.64” N 119° 27’ 20.16” W | 1,798 | sandy, gravelly, brown, little moisture and organic matter |
| **Northern area** |  |  |  |
| T-40-4200-1 | 35° 10’ 49.08” N 119° 35’ 49.20” W | 2,018 | fine particulate, light brown, little moisture |
| T-40-4200-2 | 35° 11’ 04.92” N 119° 36’ 03.24” W | 1,827 | fine particulate, brown, little moisture, and organic matter |
| T-40-4200-3 | 35° 11’ 20.04” N 119° 36’ 19.44” W | 1,864 | fine particulate, brown, little moisture, and organic matter |
| T-40-4200-4 | 35° 11’ 48.48” N 119° 36’ 48.96” W | 1,883 | very fine particulate, dry, light tan |
| **T-40-4200-5** | 35° 12’ 21.24” N 119° 37’ 00.12” W | 2,720 | fine particulate, dry, brown, organic matter |
| **T-40-4200-6** | 35° 12’ 09.00" N 119° 37’ 38.28” W | 2,001 | fine particulate, dry, brown, organic matter |
| T-40-4200-7 | 35° 11’ 53.52” N 119° 38’ 02.04” W | 2,441 | very fine particulate, tan, little moisture, and organic matter |
| T-40-4200-8 | 35° 11’ 58.92” N 119° 38’ 33.14” W | 2,467 | very fine particulate, tan, little moisture, and organic matter |
| T-40-4200-9 | 35° 12’ 02.52” N 119° 39’ 13.32” W | 2,244 | very fine particulate, tan, little moisture, and organic matter |
| T-40-4200-10 | 35° 12’ 22.68” N 119° 39’ 56.16” W | 2,359 | very fine particulate, tan, little moisture, and organic matter |
| T-40-4200-11 | 35° 12’ 37.08” N 119° 40’ 04.44” W | 2,421 | off-road, fine particulate, dry, brown, gravelly, organic matter |
| T-40-4200-12 | 35° 12’ 43.56” N 119° 40’ 27.12” W | 2,510 | dry, brown, rocky, organic matter |
| T-40-4200-13 | 35° 13’ 05.52” N 119° 40’ 30.00” W | 2,516 | brown, little moisture and organic matter |
| T-40-4200-14 | 35° 13’ 29.64” N 119° 40’ 27.48” W | 2,369 | off-road, fine particulate, brown, little moisture, and organic matter |
| T-40-4200-15 | 35° 13’ 42.24” N 119° 40’ 25.68” W | 2,283 | fine particulate, ochre, little moisture, and organic matter |
| **T-4300-1** | 35° 11’ 24.00” N 119° 34’ 42.96” W | 1,457 | sandy, dry, light brown, organic matter |
| T-4300-2 | 35° 11’ 13.20” N 119° 34’ 50.16” W | 1,562 | sandy, little moist, light brown |
| T-4300-3 | 35° 10’ 57.00” N 119° 34’ 49.44” W | 1,640 | sandy, dry, brown |
| T-4300-4 | 35° 10’ 41.52” N 119° 34’ 52.68” W | 1,755 | sandy, little moist, light brown |
| T-4300-5 | 35° 10’ 33.96” N 119° 35’ 23.64” W | 1,883 | powdery, dry, light brown |

**Supplementary Table S4.** Comparing electrical conductivity (EC) values for different soil types. Tukey test results of re-transformed data.

| **Sites compared** |  |  |  |
| --- | --- | --- | --- |
| **High Site** | **Low Site** | **EC difference** | ***p*** |
| **Low EC Group Comparison** |  |  |  |
| Elkhills-Welport | Littlesignal-Cochora | 0.251 | 0.91 |
| **High EC Group Comparisons** |  |  |  |
| Beam-Panoza-Hillbrick | Xeric-Torriorthents-Badlands | 0.219 | 1 |
| Xeric-Torriorthents-Badlands | Pyxo-Cochora and PC Badlands | 0.026 | 0.995 |
| Beam-Panoza-Hillbrick | Pyxo-Cochora and PC Badlands | 0.245 | 0.947 |
| **Cross-group Comparisons** |  |  |  |
| Pyxo-Cochora and PC Badlands | Elkhills-Welport | 0.172 | 0.627 |
| Xeric-Torriorthents-Badlands | Elkhills-Welport | 0.198 | 0.635 |
| Beam-Panoza-Hillbrick | Elkhills-Welport | 0.417 | 0.241 |
| Pyxo-Cochora and PC Badlands | Littlesignal-Cochora | 0.423 | 0.204 |
| Xeric-Torriorthents-Badlands | Littlesignal-Cochora | 0.449 | 0.262 |
| Beam-Panoza-Hillbrick | Littlesignal-Cochora | 0.668 | 0.0551 |

**Supplementary Table S5.** Descriptive statistics with results of one-way ANOVAs by year for pH and EC (mS/cm) for each sampling year for presence/absence of the pathogen (**significant difference).

| **pH** | | | | | | | | | | |
| --- | --- | --- | --- | --- | --- | --- | --- | --- | --- | --- |
| ***Coccidioides* present** | | | | ***Coccidioides* absent** | | |  |  |  |  |
| **Year** | **mean** | **s** | **n** | **mean** | **s** | **n** | **F** | **df** | ***p*** | **% Positive** |
| 2018 | 7.56 | 0.399 | 14 | 7.54 | 0.521 | 22 | 0.015 | 1,34 | >0.9 | 38.9 |
| 2019 | 7.97 | 0.128 | 5 | 7.86 | 0.448 | 18 | 0.257 | 1,21 | >0.6 | 21.7 |
| 2021 | 7.55 | 0.264 | 13 | 7.83 | 0.254 | 28 | 11 | 1,39 | <0.005** | 31.7 |
| **Electrical Conductivity (EC)** | | | | | | | | | | |
| ***Coccidioides* present** | | | | ***Coccidioides* absent** | | |  |  |  |  |
| **Year** | **mean** | **s** | **n** | **mean** | **s** | **n** | **F** | **df** | ***p*** | **% Positive** |
| 2018 | 1.22 | 0.858 | 14 | 0.658 | 0.59 | 22 | 5.84 | 1,34 | <0.05 | 38.9 |
| 2019 | 0.778 | 0.692 | 5 | 1.16 | 1.13 | 18 | 0.128 | 1,21 | >0.7 | 21.7 |
| 2021 | 0.412 | 0.327 | 13 | 0.224 | 0.224 | 28 | 3.04 | 1,39 | >0.05 | 31.7 |
